# Frequency, geographical distribution, clinical characteristics, antivenom utilisation and outcomes of King Cobra (*Ophiophagus hannah*) bites in Malaysia

**DOI:** 10.1371/journal.pntd.0012359

**Published:** 2024-07-25

**Authors:** Asyhok Renault, Vera Effa Rezar Frederic Ng, Wan Chee Goh, Muhammad Nadzmi Hadi Abd Hamid, Annuar Muhammad Zuljamal Osman, Ruth Sabrina Safferi, Zainalabidin Mohamed@Ismail, Ahmad Khaldun Ismail

**Affiliations:** 1 Department of Emergency Medicine, Faculty of Medicine, Universiti Kebangsaan Malaysia, Kuala Lumpur, Malaysia; 2 Emergency and Trauma Department, Hospital Raja Permaisuri Bainun, Ipoh, Perak, Malaysia; 3 Emergency and Trauma Department, Hospital Tengku Ampuan Afzan, Kuantan, Pahang, Malaysia; 4 Hospital Canselor Tuanku Muhriz, Jalan Yaacob Latif, Bandar Tun Razak, Kuala Lumpur, Malaysia; Universidade Nilton Lins, BRAZIL

## Abstract

Snakebite envenomation remains an important, yet a neglected public health issue in most tropical and subtropical countries. Underdeveloped medical infrastructure, suboptimal medical services, poor documentation and failure to make snake-related injury a mandatory notifiable disease are important contributing factors. The King Cobra *(Ophiophagus hannah*) is a medically significant species encountered in Malaysia however, there have been few publications from the clinical perspective. The objectives of this study were to determine the frequency of King Cobra related injuries, geographical distribution, clinical presentation, type and frequency of antivenom utilization and the management outcome. This is a cross-sectional study of confirmed King Cobra related injuries consulted to Remote Envenomation Consultation Services (RECS) from 2015 to 2020. Data were extracted from the RECS database and descriptively analyzed. A total of 32 cases of King Cobra bite were identified. Most cases were from Peninsular Malaysia with the most frequent from the state of Pahang (*n* = 9, 28.1%). Most patients got bitten while attempting to catch or play with the snake (68.8%). Signs and symptoms of envenomation were documented in 24 (75.0%) cases and the most frequent systemic manifestation was ptosis (*n* = 13, 40.6%). Tracheal intubation and ventilatory support were required in 13 (40.6%) patients. Antivenom was administered to 22 (68.8%) patients with most (25.0%) receiving 10 vials (1 dose). The commonest antivenom used was monospecific *King Cobra* antivenom (50.0%) from Thai Red Cross. There was one death documented due to complications from necrotizing fasciitis and septicemia. Public awareness of the dangers and proper handling of King Cobras needs to be emphasised. Timely administration of the appropriate antivenom is the definitive treatment and leads to favorable outcomes.

## Author summary

King Cobra is the longest venomous snake in the world and is widely distributed in Peninsular and East Malaysia. There is lack of documentation and publication of King Cobra bite injuries in Malaysia. This may lead to poor awareness of the significance and the severity of envenomation from this species. There are two types of antivenom used in Malaysia for treating King Cobra envenomation, namely Queen Saovabha Memorial Institute (QSMI), Thai Red Cross King Cobra antivenom and QSMI neuropolyvalent antivenom. There were no previous publications on the clinical outcomes of the utilisation of these antivenom for King Cobra envenomation in Malaysia. This study analysed confirmed King Cobra bite injuries consulted to Remote Envenomation Consultancy Services (RECS) from 2015–2020. The RECS consultation log was analysed for the frequency, geographical distribution, clinical features, management and outcome. This study provides verified and reliable information that can be used in the future for developing health policy and clinical management guidelines.

## Introduction

**Fig 1:**
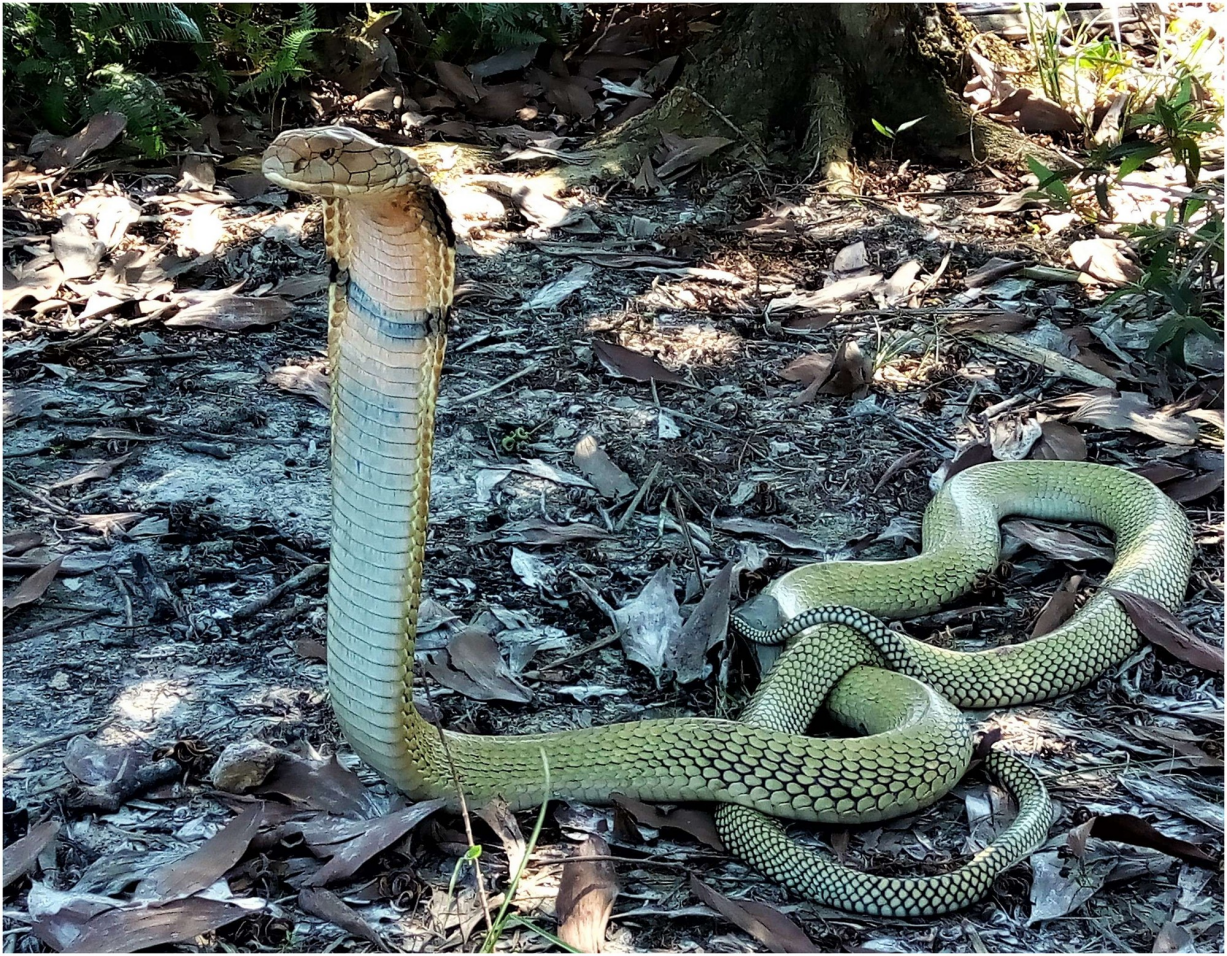
The morphological feature of a Malaysian King Cobra, *Ophiophagus hannah*. Image credit: A.K. Ismail.

Snake bite envenomation remains an important, yet a neglected public health issues in many parts of the world [1,2–5]. Although, the majority of snake species in Malaysia are harmless and non-venomous, bite envenomation from King Cobra, *Ophiophagus hannah* (Fig 1) may result in significant morbidity and mortality [3,6]. King Cobra envenomation is relatively uncommon, and usually involves those free handling snakes [7]. However, due to rapid deforestation, urbanisation, and human encroachments into snakes’ habitats, the risks of bite envenomation may also increase [8,9]. Many rescue efforts took place in residential area and homes. King Cobra is also frequently captured and traded as pets or entertainment.

King Cobra is the world’s longest venomous snake and widely distributed throughout Southeast Asia including Malaysia, Southern China, and parts of the Indian subcontinent [9–13]. The term *Ophiophagus* is derived from its tendency to prey on other snakes. King Cobra is typically timid, and despite its intimidating size, it avoids confrontation with humans. However, when threatened, the King Cobra can become aggressive and tends to ‘bite and hold’ for an extended period to deliver large amounts of venom [9,12,13]. The major constituents of King Cobra venom are polypeptide neurotoxins, haemorrhagic and nonhemorrhagic proteases, phospholipases A2 (PLA2), L-amino acid oxidases (LAAO), and alkaline phosphomonoesterases [12,14,15]. The severity of envenomation and clinical manifestation depends on the duration of bite and the amount of venom injected. The major neurotoxic component affects the post-synaptic neuromuscular junction, causing descending paralysis and fatality may result from asphyxiation [3,10,12]. King Cobra from various geographical areas displays distinct morphological phenotypes from one another, proposing the possibility of taxonomic and venom divergence within this monotypic species [12,13]. Systemic envenomation usually requires mechanical ventilation and the appropriate antivenom administration [3,12]. There are two types of antivenom used in Malaysia for treating King Cobra envenomation, namely monospecific King Cobra antivenom and neuropolyvalent antivenom manufactured by Queen Saovabha Memorial Institute (QSMI), Thai Red Cross in Thailand [3,16].

Snakebite and envenomation management in Malaysia is supported by the Remote Envenomation Consultancy Services (RECS) [16]. RECS is a specialised online risk management support system provided by Emergency Physicians who are members of the College of Emergency Physician special interest group in Clinical Toxinology and the Malaysian Society on Toxinology (MST). The principle objective of RECS is to enhance a favourable outcome by optimizing and advocating appropriate treatment modalities at every level of clinical management. RECS consultation provides a comprehensive diagnosis for each case. Details acquired during RECS consultation follows the standards provided in the Malaysian Ministry of Health Snakebite Management Guidelines (2017). The species confirmation was verified by RECS consultants based on the specimen brought to the hospital or the picture of the actual specimen taken and documented in each case diagnosis in the RECS consultation log. The data from each consultation is compiled into individual case files and deposited into a systematic registry called Malaysian Biodiversity Information System (MyBIS) Toxinology module at Forest Research Institute Malaysia (FRIM), Ministry of Natural Resources and Sustainability. The database is maintained under Malaysian Administrative Modernisation and Management Planning Unit (MAMPU), Prime Minister’s Department.

There is limited information on King Cobra bite envenomation in Malaysia which may also reflects poor awareness and documentation. The objectives of this study were to determine the frequency, geographical distribution, clinical profile, treatment modalities and outcome following King Cobra bites consulted to RECS.

## Methods

### Ethics statement

Data was collected following the approval of institutional research ethics committee Faculty of Medicine, National University of Malaysia (UKM FF-2021-241) and RECS coordinator.

This is a retrospective cross-sectional study of verified King Cobra bites in Malaysia consulted to RECS from January 2015 to December 2020. A universal sampling method was used whereby all case details were collected from RECS consultation log and case record. Only confirmed cases of King Cobra species were included. The documentation process involved filing of individual cases consulted to RECS into the standardised data collection sheet. Various information such as demography, incident location, clinical history, clinical presentation, snake species identification, clinical manifestation and progression, investigation, treatment and outcome during the consultation were extracted. The data for each year was documented separately in the data collection sheet and analysed using Statistical Package for the Social Sciences (SPSS) version 24.0. All data collected from RECS database were kept anonymous and confidential.

## Results

There was a total of 4901 RECS consultations with 4109 (83.8%) SRI in the period of 6 years from 2015–2020. A total of 32 (0.8%) cases were confirmed King Cobra bite injuries. Most of the patients were male (93.8%) and in the age group 16–30 years old (43.8%) (Table 1). The mean age was 34.58 years old (SD ±18.5). The youngest victim was 1 year old and the oldest was 77 years old. Most cases involved Malaysians (93.8%) with Malays (87.5%) being the most common ethnicity involved. Most incidences were non-occupational related (65.6%) with fire and rescue services (21.9%) being the most common occupation.

**Table 1:** Sociodemographic features of King Cobra bite injuries patients in Malaysia consulted to RECS from 2015 to 2020.

Variables		n (%)
**Age**	0–15	2 (6.3%)
	16–30	14 (43.8%)
	31–45	7 (21.9%)
	46–60	4 (12.5%)
	>60	4 (12.5%)
	Undocumented	1 (3.1%)
**Ethnicity**	Malay	28 (87.5%)
	Indigenous	1 (3.1%)
	Others (non-Malaysian)	2 (6.3%)
	Undocumented	1 (3.1%)
**Occupation**	Fire & Rescue	7 (21.9%)
	Civil Defence	3 (9.4%)
	Agricultural worker	1 (3.1%)
	Non-occupational related	21 (65.6%)

**Fig 2:**
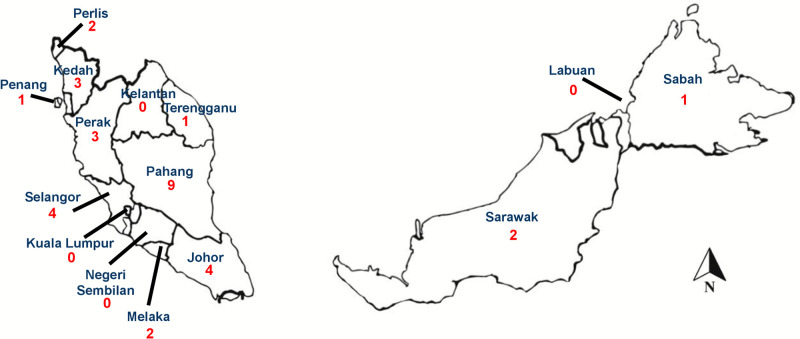
Geographical distribution of King Cobra bite consulted to RECS in each state of Peninsular Malaysia and East Malaysia from 2015–2020. Base map and data from OpenStreetMap and OpenStreetMap Foundation. (OpenStreetMap contributors, https://www.openstreetmap.org/#map=6/4.226/108.237).

The majority of RECS consultation for King Cobra bite was from the state of Pahang (n = 9, 28.1%) followed by the states of Selangor and Johor (Fig 2).

**Fig 3:**
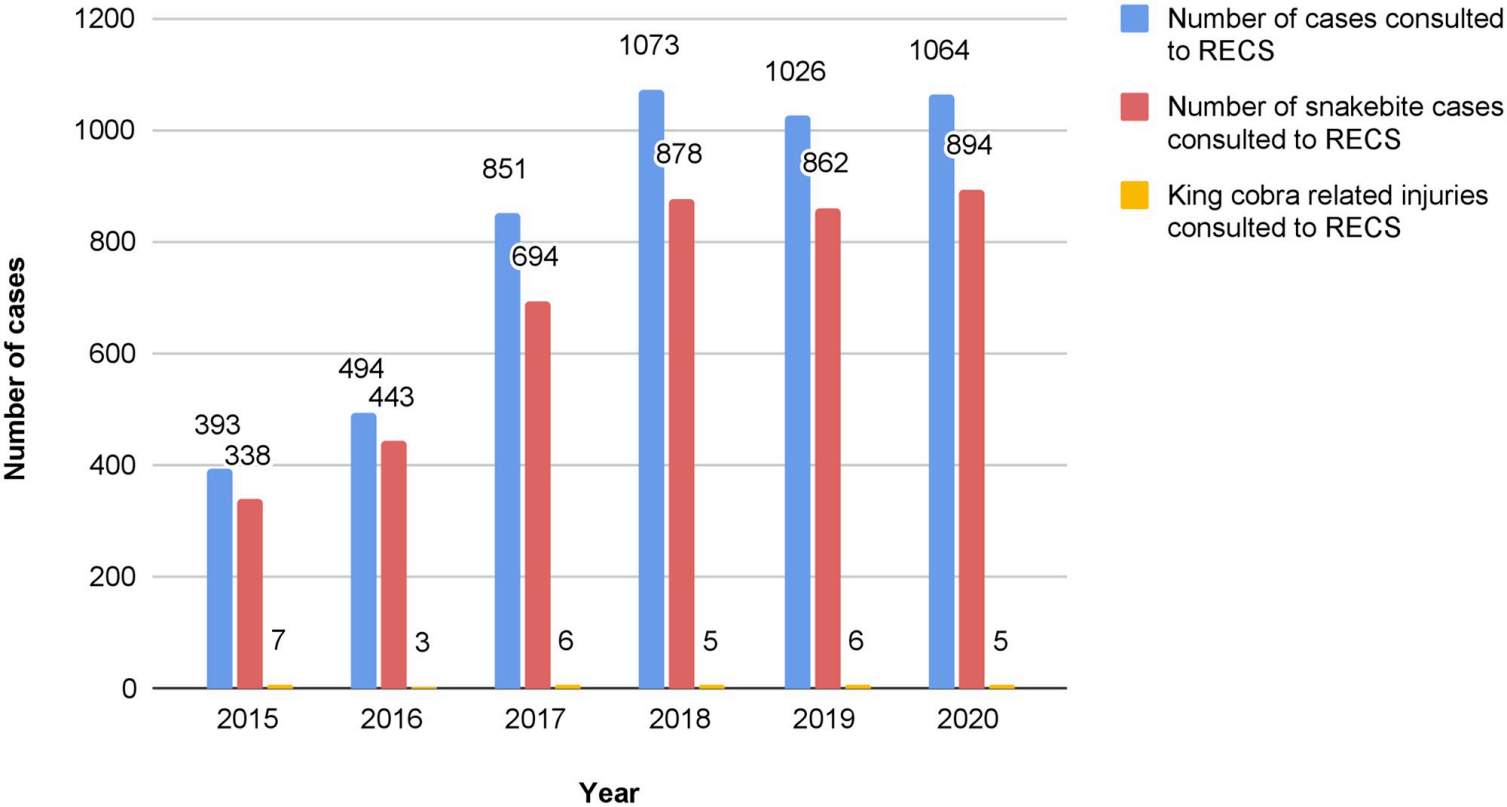
Number of RECS consultations, frequency of SRI and King Cobra related injuries consulted to RECS from 2015 to 2020.

The highest frequency of incident was in 2015 (*n* = 7, 21.9%) and the lowest was in 2016 (*n* = 3, 9.4%) (Fig 3). Most incidents occurred in the months of January, April and June with five cases occurring per month (15.6%). The lowest number of cases occurred in the months of July, August and October with one case occurring per month (3.13%). Most cases happened between noon and late afternoon (*n* = 14, 43.8%) (Table 2). Majority of patients got bitten while attempting to catch the snake (*n* = 14, 43.8%) followed by playing with snakes (*n* = 8, 25.0%). Upper limb is the most affected anatomical region of the body (*n* = 20, 62.50%). Most patients presented to the emergency department within 30 minutes following the incident (*n* = 6, 18.8%), however there were a significant number where the time interval was unable to be determined (*n* = 14, 43.8%).

**Table 2:** Total number of King Cobra related injuries per month over the 6 years study period, time and activity during incident, anatomical region affected and the time interval between incident and hospital arrival.

Variables	n (%)
**Time of incidence**	
0100–0600	0
0601–1200	7 (21.9)
1201–1800	14 (43.8)
1801–0000	11 (34.4)
**Activity during incident**	
Catching	14 (43.8)
Playing with snake	8 (25.0)
Rescue handling	7 (21.9)
Harming	1 (3.1)
Fishing	1 (3.1)
Undetermined	1 (3.1)
**Anatomical distribution**	
Upper limb extremity	20 (62.5)
Lower limb extremity	9 (28.1)
Head and neck	3 (9.4)
**Time interval between incident and hospital arrival**	
< 1 hour	11 (34.4)
1 to 4 hours	6 (18.8)
>4 hours	1 (3.1)
Undocumented	14 (43.8)

The majority of patients did not initiate any form of first aid prior to hospital arrival (*n* = 17, 53.1%). Tourniquet was the most common method of first aid used (*n* = 7, 21.9%) (Table 3). Most patients presented with signs of envenomation indicated for antivenom (*n* = 22, 68.8%). The most frequent local effects were bite/puncture mark (*n* = 31, 96.9%) and oedema (*n* = 23, 71.9%). The majority of cases documented mild to moderate pain (28.1%). Nausea and vomiting (*n* = 4, 12.5%) were the most frequent general symptoms. Shortness of breath/respiratory distress and dizziness (*n* = 7, 21.9%) were the most common cardiorespiratory symptoms and ptosis (*n* = 13, 40.6%) was the most frequent early neurological sign. The most frequent medical treatment was antivenom and analgesia (*n* = 22, 68.8%). Intubation and ventilator support were required in 13 (40.6%) patients. Wound debridement was performed in five (15.6%) patients, and fasciotomy in four (12.5%) patients.

**Table 3:** The first aid performed, clinical manifestation, medical management and surgical intervention of King Cobra bite.

Variables	n (%)
**First aid done prior to hospital arrival**	
None	17 (53.1)
Tourniquet application	7 (21.9)
Application of topical herbs	1 (3.1)
Incision of bite wound	1 (3.1)
Sucking of bite wound	1 (3.1)
Others	10 (31.3)
**General effects**	
Nausea	4 (12.5)
Vomiting	4 (12.5)
Diaphoresis	3 (9.4)
Drowsiness	2 (6.3)
Body weakness	1 (3.1)
Others	3 (9.4)
**Local effects**	
Bite/puncture mark	31 (96.9)
Oedema/swelling	23 (71.9)
Rapid extension of swelling	15 (46.9)
Mild to moderate pain	14 (43.8)
Severe pain	9 (28.1)
Swelling > half of bitten limb within 48 hr	8 (25.0)
Tissue necrosis	6 (18.8)
Tender enlarged lymph nodes	6 (18.8)
Bleeding	5 (15.6)
Blister/bullae	4 (12.5)
Cyanosed/discolouration of bitten limb	2 (6.3)
Pulselessness of bitten limb	2 (6.3)
Others	8 (25.0)
**Cardiorespiratory effects**	
Dizziness	7 (21.9)
Shortness of breath/respiratory distress	7 (21.9)
Hypotension (BP<90/60)	5 (15.6)
Abnormal ECG	1 (3.1)
Signs of shock	1 (3.1)
Fainting	1 (3.1)
Others	2 (6.3)
**Neurotoxic effects**	
Ptosis	13 (40.6)
Poor respiratory effort	6 (18.8)
Speaking difficulty/aphonia	2 (6.3)
Dilated pupils	2 (6.3)
Absence of light reflex	2 (6.3)
Cranial nerve palsy	1 (3.1)
Broken neck syndrome	1 (3.1)
External ophthalmoplegia	1 (3.1)
Limbs paralysis	1 (3.1)
Others	10 (31.3)
**Medical Management**	
Analgesia	22 (68.8)
Antivenom	22 (68.8)
Intubation and ventilatory support	13 (40.6)
Anti-tetanus toxoid (ATT)	9 (28.1)
Antibiotics	12 (37.5)
Steroids	7 (21.9)
Inotropes	7 (21.9)
Oxygen therapy	3 (9.4)
Blood tranusion	1 (3.1)
Others	5 (15.6)
**Surgical Intervention**	
Wound debridement	5 (15.6)
Fasciotomy	4 (12.5)
Amputation of limb	1 (3.1)
Others	1 (3.1)

Among the 22 (68.8%) patients who received antivenom therapy, most (*n* = 11, 50.0%) were initiated in the emergency department (Table 4). The majority (*n* = 8, 36.4%) received 10 vials of antivenom. The mean number of vials given was 14. Most patients (63.6%) required more than one course of antivenom administration. *King Cobra* monospecific antivenom was the most frequent type of antivenom used (*n* = 11, 50.0%). The majority (*n* = 16, 72.7%) did not develop reactions or complications from antivenom therapy. Two patients (33.3%) required adrenaline administration. Two patients (n = 2, 9.1%) received inappropriate antivenom therapy. In 2015, a patient was given two vials of *Naja Kaouthia* antivenom (NKAV) and four vials of Indian polyspecific antivenom. In 2020, a patient was given 10 vials of NKAV. Both cases received improper antivenom prior to RECS consultation. However, these errors were rectified by RECS and appropriate antivenom was administered.

**Table 4:** The frequency of antivenom usage, venue of initiation, number of vials administered, course of therapy, types of antivenom used, complications and management outcomes of King Cobra bite envenomation.

Variables	n (%)
**Antivenom usage**	
Yes	22 (68.8)
No	10 (31.3)
**Initiation of antivenom**	
District hospital	10 (45.5)
Tertiary hospital (emergency department)	11 (50.0)
Tertiary hospital (ward/ICU)	1 (4.5)
**Total number of vials given**	
5	1 (4.5)
8	4 (18.2)
10	8 (36.4)
15	1 (4.5)
20	4 (18.2)
25	3 (13.6)
30	1 (4.5)
**Course of antivenom given (n = 22)**	
Single course	8 (36.4)
Repeat course	14 (63.6)
**Type of antivenom administered**	
**King Cobra** antivenom (OHAV) only	11 (50.0)
Neuropolyvalent antivenom (NPAV) only	3 (13.6)
Combination (OHAV and NPAV)	8 (36.4)
Inappropriate	2 (9.1)
**Complications related to antivenom (n = 6)**	
Antivenom-induced anaphylaxis	1 (4.5)
Antivenom-induced allergic reaction	5 (22.7)
**Treatment given for antivenom related complications**	
Adrenaline	2 (33.3)
Hydrocortisone	6 (100.0)
Chlorpheniramine maleate	4 (66.7)
**Complications related to injury**	
Presumed compartment syndrome	4 (12.5)
Sepsis	1 (3.1)
Necrotizing fasciitis	2 (6.3)
Multiorgan failure	1 (3.1)
**Hospital disposition**	
Admission to ward	14 (43.8)
Admission to critical care unit	13 (60.3)
Discharge from emergency department	1 (3.1)
Discharge against medical advice	1 (3.1)
Absconded	1 (3.1)
Undocumented	2 (6.3)
**Length of stay**	
1 day	5 (18.5)
2–3 days	8 (29.6)
4–5 days	7 (25.9)
6–10 days	4 (14.8)
>10 days	1 (3.7)
Undocumented	7 (21.9)
**Final outcome**	
Discharged well	20 (62.5)
Discharged with permanent morbidity/injury	4 (12.5)
Discharged against medical advice / absconded	2 (6.3)
Death	1 (3.1)
Undocumented	5 (15.6)

The commonest complication encountered was presumed compartment syndrome (*n* = 4, 12.5%) with 27 (84.4%) patients required admission to either emergency department or critical care unit. Most patients (*n* = 8, 29.6%) were hospitalized for two to three days. The longest duration of hospitalization was 14 days. The median length of stay was 3 (IQR 1–5) with the longest stay of 14 days. The majority (*n* = 20, 62.5%) of patients were discharged well with one death documented.

## Discussion

The frequency of King Cobra related injuries consulted to RECS remains below ten cases per year despite the increasing annual rate of SRI. The trend may have been influenced by the continuous efforts on safety awareness campaigns. Another important factor that may have minimized the rate of injuries from King Cobra is the restriction of private ownership licences for captive wildlife by Department of Wildlife and National Parks Peninsular Malaysia (PERHILITAN) since 2016.

King Cobra bites mainly in defence when provoked or accidentally harmed [3] and most non-occupational related patients sustained the bite while trying to capture it. Some injuries occurred when they were playing with a wild-caught snake or while handling their pet snakes. The occupational related injuries were mainly from poor handling by rescuers while securing snakes into bags or containers. It was not established whether the rescuers were wearing appropriate protective gears. However, there is a standard protocol for appropriate equipment and protective gears during the rescue effort. There is a need for proper training and to follow established protocol. Regular training should be conducted by experienced personnel.

Most King Cobra related injuries affect the upper limbs. The upper extremities are commonly associated as illegitimate bites from activities including handling, harming, catching or playing captive snakes [6,17,18,19,20]. However, this study also found bite injuries involving lower limbs occurred while intentionally interacting with the King Cobra. An example was a 69-year-old gentleman purposefully allowed himself to be bitten on his right knee with the intention of achieving immunity (mithridatism) from the venom.

Majority of patients in this study seek medical treatment within the first 4 hours of bite incident. Various public awareness programs regarding snakebites conducted by RECS may have played a positive role. The easy access to quality services in the Malaysian healthcare system has encouraged patients to decide for the appropriate seeking behaviour following a snakebite injury. Early access to healthcare services allows for timely and appropriate treatment for the optimal outcome.

There are limited published data on the envenomation effects of King Cobra bite [7,10,15,21–24]. Due to the constraint on existing studies, clinical presentations of these case reports were utilized to compare the clinical manifestations of the patients in this study. The two most common general effects following a bite are nausea and vomiting. The cardiorespiratory manifestations frequently seen are shortness of breath, dizziness and episodes of hypotension. Observations from the case reports and from this study suggests that these three cardiorespiratory symptoms are commonly associated with bites at presentation in comparison to cardiac arrhythmias, abnormal ECG, cardiac arrest and pulmonary oedema. Neurotoxicity is the most common finding with ptosis being the most frequent early sign. This is comparable to the published case reports. Rapid progression of neurotoxic manifestation often contributes to events leading to fatality. All patients in this study who presented with early neurotoxic signs benefited from a timely antivenom administration. This is likely to have resulted in better outcome for patients.

In most instances, the King Cobra ‘bite and hold’ its prey allowing for larger volume of venom to be injected [9]. A single bite is able to deliver an average of 1 gram (dry weight) of venom [15]. Patients commonly complained of immediate pain at the bite site which worsens with the development of progressive swelling. Rapid extension of painful swelling is seen in nearly half of the patients in this study. The other features that contributed to pain progression were related to the amount of tissue necrosis and lymphadenitis presented with enlarged tender lymph nodes [21–28]. The venom also contains L-amino acid oxidase (LAAOs). Pain and local tissue damage are believed to be caused by this enzyme [15]. Most patients in this study developed both local and systemic effects compared to only local envenomation.

This study revealed that more than half of patients did not receive any first aid with mixed findings on the prognosis of the wound. Several methods of inappropriate first aid was still widely practiced. Tourniquet application is common [29,30]. Others attempted to massage or rinsing with water. Some squeeze, pinch, incise, suck and apply topical herbs to the bitten area. Patients who received first aid treatment with tourniquet were identified to have more severe local swelling [31–34]. These inappropriate practices may be related to various deep rooted indigenous and cultural beliefs.

Management of King Cobra bite victims requires a multidisciplinary approach, which include medical and surgical interventions. Even though antivenom therapy was the most common treatment received, a few patients still required mechanical ventilation and underwent surgical debridement or amputation. This study suggests that patients bitten by King Cobra have a high risk for definitive airway management. The neurotoxic venom leads to defective transmission at the neuromuscular junction which gives rise to a neuromuscular paralysis causing respiratory failure [35]. Physicians attending to patients who sustained a King Cobra bite should anticipate early endotracheal intubation.

Steroid is not classified as a standard care of acute SRI management. Corticosteroids usage does not have an added advantage to the outcome in snake bite victims [2,36]. In this study, steroid was initiated prior to RECS consultation in health clinics and district hospital settings. This could be due to the lack of experience and knowledge in managing SRI. The utilization of steroids as prophylaxis to prevent adverse effects from antivenom administration is well defined [2, 30].

Antivenom therapy is the definitive treatment in King Cobra envenomation. Signs and symptoms of envenomation were observed in most cases in this study and most of them have successfully received antivenom therapy. Those having mild non-progressing local envenomation were not given antivenom. The administration of antivenom was advocated as per the Clinical Practice Guideline when either one or more of these neurotoxic signs were noticed; paradoxical respiration, ptosis, fixed dilated pupils, absence of light reflex, external ophthalmoplegia, paralysis of neck flexor muscles, difficulty in swallowing, cranial nerve palsy, regurgitation and aphonia [16]. The antivenom therapy was also commenced in presence of any one of the local envenomation signs which include local swelling involving more than half of the bitten limbs within 48 hours of bite in absence of tourniquet application / swelling after bites on the digits, rapid extension of swelling beyond wrist or ankle within few hours of bite on either hands or feet and development of an enlarged tender lymph node draining the bitten area. The use of point-of-care ultrasound (POCUS) to measure the rate of proximal progression of the oedema can help to determine the indication and response to antivenom and prevent wastage [37]. Antivenom therapy was either initiated at the district hospital or the emergency department of a tertiary hospital. This is achievable because most district hospitals are equipped with the appropriate antivenom and clinical support via remote consultation with RECS or medical toxicologists. Other contributing factors in timely intervention with antivenom therapy are early presentation to the emergency department and prompt detection of signs and symptoms of envenomation.

Prior to 2012, most Malaysian hospitals had been stocked with inappropriate antivenom from India. Antivenom procurement in Malaysia had a major revamp in 2012 following a severe King Cobra envenomation in the state of Pahang. Appropriate antivenoms from QSMI Thailand were subsequently included in the clinical management guidelines. The procurement of antivenom is based on the need of each individual hospital. In this study, half of the patients who received antivenom therapy were given monospecific QSMI *Ophiophagus hannah* antivenom (OHAV). Most patients whose antivenom therapy was initiated at a district hospital were given QSMI Neuropolyvalent antivenom (NPAV) whereas patients in tertiary hospital received OHAV. The differences in initial antivenom selection are probably due to district hospitals having limited stocks of OHAV. The preference of antivenom selection is decided based on its availability and suitability of the antivenom for the healthcare facility.

The majority of those who received antivenom required additional doses. A preclinical study in 2019 reported an average of 17 vials of Thai King Cobra monovalent antivenom is needed to neutralize the Malaysian King Cobra venom [12]. The average antivenom used in this study was 14 vials which corresponds to the preclinical study. An envenomation related to King Cobra bite is likely to require greater quantities of antivenom in comparison to other species [38]. The required dosage and preferred courses of antivenom therapy is highly dependent on individual patient’s clinical response. An estimation of 10–30 vials is therefore recommended to be on standby for each King Cobra envenomation in Malaysia [12,15].

The two common adverse effects seen in this study are allergic reactions and antivenom induced anaphylaxis. In this study, the term antivenom induced allergic reaction was used for the mild reaction which manifested as urticaria rash with generalized itching or flushing or mucosal oedema following the administration of an antivenom [39–44]. On the contrary, the term antivenom induced anaphylaxis is used for more severe reactions which demonstrate two or more signs and symptoms that occur following an antivenom administration. These include either skin and or mucosal involvement, respiratory compromise, hypotension or cardiovascular collapse and persistent gastrointestinal cramps or vomiting [41–44]. It appears that the risk of antivenom induced complications in Malaysia is low. In cases which involved adverse reactions, patients were given intravenous steroids and antihistamine. Severe reaction also received adrenaline. It is worth noted that treatment was only initiated after complications arise and not as prophylaxis. Although there is lack of evidence to support prophylaxis steroid and antihistamine in reducing adverse effects, prophylaxis adrenaline could be useful for certain situations with regards to the quality of antivenom and allergy history of patients [30].

Surgical intervention is less commonly used than medical therapy in managing complications related to King Cobra envenomation. In this study, several patients underwent wound debridementand one patient had his limb amputated. Four patients with presumed compartment syndrome were prematurely subjected to fasciotomy without an intra-compartmental pressure measurement. Two of them were treated before consulting RECS and the decision for the other two was made against RECS recomendation to delay surgical intervention until after antivenom optimization. It is frequently observed that local swelling following snakebite envenomation may mimic compartment syndrome [16, 45–47]. Timely appropriate and adequate antivenom therapy is the preferred choice of treatment. Fasciotomy should only be reserved for significantly raised intracompartmental pressure. The intra-compartmental pressure measurement can be determined by a few methods which include handheld manometer (i.e. Stryker device), simple needle manometer system or slit catheter technique. Capillary blood flow is often compromised when the intra-compartmental pressure level rises within 25–30 mmHg of mean arterial pressure (MAP). Consecutively, ischemia occurs when the tissue pressure approaches diastolic blood pressure (DBP). The delta pressure is defined as DBP minus measured intra-compartmental pressure, and often fasciotomy is considered when the delta pressure is lower than 20-30mmHg [45]. The World Health Organization recommends fasciotomy only in cases with clear clinical evidence of compartment syndrome and a recorded value of intra-compartmental pressure of > 40mmHg [2].

It is however important to have a high index of suspicion for compartment syndrome in patients who developed intramuscular swelling [37,37]. In this study, an urgent ultrasound for one patient who exhibited all clinical signs of compartment syndrome revealed only subcutaneous tissue thickening and oedema. The muscle layers were preserved with no evidence of intramuscular collection. This patient was initially planned for fasciotomy by the orthopaedic surgeon however, following RECS recommendations it was averted. The patient had full recovery following antivenom therapy. In cases of presumed compartment syndrome following snake bites, an ultrasound often reveals that the primary site of swelling is confined to subcutaneous tissue. Point of care ultrasonography (POCUS) can be useful as a non-invasive adjunct for bedside monitoring of muscle compartment status [45,46]. Local swelling and the rate of proximal progression can be documented as well as measuring the diastolic retrograde arterial flow (DRAF) in the affected artery.

Other infrequent complications resulting from a King Cobra bite include necrotizing fasciitis, wet gangrene, disseminated intra vascular coagulation (DIVC) and renal failure. Most of these conditions were rarely encountered because majority of patients benefited from early empirical administration of antivenom therapy. In this study, two were diagnosed with necrotizing fasciitis and one wet gangrene. All three patients obtained positive microbial culture. The first patient with necrotizing fasciitis had *Pseudomonas sp*. isolated from tissue culture and the latter’s blister fluid culture grew *Aeromonas sp*. Tissue culture in the patient with wet gangrene grew *Enterobacter aerogenes*. *Pseudomonas* and *Aeromonas sp*. are not typical organisms causing necrotizing fasciitis [48,49]. Broad categories of organism has been identified from the oral cavity of a snake such as *Pseudomonas* sp., *Aeromonas* sp., *Morganella* sp., *Salmonella* sp., *E*. *coli*, *Enterococcus* sp., *Staphylococcus* sp. and various others [50,51]. The appropriate selection of antibiotics for these patients were then tailored based on the culture and sensitivity results.

Most patients in this study required admission either to the general ward or critical care unit (ICU) following a short stay in the emergency department after the initial antivenom treatment. Patients who are subjected to a longer hospital stay are usually those admitted to the ICU and those who developed severe complications or underwent surgical interventions. Majority of patients were discharged well however, the outcomes of two patients who requested for DAMA and absconded were not traceable from the RECS consult database. There was one death documented in 2018 following complications of necrotizing fasciitis and severe sepsis. The South Asia and Southeast Asia countries appears to record greater mortality frequency from King Cobra bite [52]. Many from the primary envenomation rather than from secondary complications. Early access to a healthcare facility and availability of appropriate antivenom coupled with good healthcare services in Malaysia may have played a crucial role for survival.

It is therefore important to promote awareness among public inclusive medical personnel on appropriate first aid measures and management of snake bite. Based on this study, we observe many shortfalls encountered in managing a snake bite. There were still misunderstanding on the indications for the appropriate antivenom and inappropriate surgical interventions. Early consultation with RECS or clinical toxicologist is therefore essential to avoid errors and to provide optimal clinical management.

## Limitations

This is a retrospective study of cases consulted only to RECS and does not reflect all King Cobra cases in Malaysia. RECS consultants are not the primary team that managed the case. Nevertheless, all diagnoses were verified by experts in the field and patient’s details were well documented with serial pictures and progress updates.

## Conclusions

This study provides crucial information regarding King Cobra bite envenoming in Malaysia. It is not uncommon with most cases consulted from the states in Peninsular Malaysia. Most bites result in local with systemic envenomation and require antivenom therapy. Mortality rate from King Cobra bite is low and was mainly due to secondarycomplications. Timely and adequate antivenom coupled with optimal wound care helps to minimise cost for unnecessary prolonged treatment and hospital stay. Free handling of King Cobra without proper training and protection should be avoided. The use of protective gear while handling or rescuing King Cobra should be made mandatory. Continuous effort to raise awareness on safety and health seeking behaviours, and medical training on snakebite management needs to be supported.

## Supporting information

S1 Data The supplementary minimal dataset of each confirmed King Cobra bite incident location, demographics, amount of antivenom usage and days of hospital stay. (DOCX)

## References

[1] Gutiérrez JM, Theakston RD, Warrell DA. Confronting the neglected problem of snake bite envenoming: the need for a global partnership. PLoS Med. 2006;3(6):e150. doi: 10.1371/journal.pmed.0030150 16729843 PMC1472552

[2] WHO. Guidelines for The Management of Snakebites 2nd ed. India: World Health Organization, Regional Office for South-East Asia; 2016.

[3] Ismail AK. Snakebite and envenomation management in Malaysia. In Toxinology: Clinical Toxinology in Asia Pacific and Africa. Netherlands: Springer; 2015. (Issue January 2015).

[4] Ismail AK, Wah TE, Das I, Vasaruchapong T, & Weinstein SA. Land Snakes Of Medical Significance In Malaysia. 3rd ed. Malaysia: Forest Research Institute Malaysia (FRIM); 2022.

[5] Das I, Ahmed N, Liat LB. Venomous terrestrial snakes of Malaysia: Their identity and biology. Clinical Toxinology in Asia Pacific and Africa, Toxinology. 2013;1–5.

[6] Chew KS, Khor HW, Ahmad R, Rahman NH. A five-year retrospective review of snakebite patients admitted to a tertiary university hospital in Malaysia. Int J Emerg Med. 2011 Jul 13;4:41. doi: 10.1186/1865-1380-4-41 21752254 PMC3143095

[7] Ismail AK, Bahrudin MF, Chun IS, Tat BY, & Abdul Hamid MF. Locked-in syndrome following a king cobra (Ophiophagus hannah) envenomation. Medicine and Health. 2017; 12: 357–362.

[8] Gowtham YJ, Mahadeswaraswamy YH, Girish KS, & Kemparaju K. Cross-reactivity and neutralization of Indian King Cobra (Ophiophagus hannah) venom by polyvalent and monovalent antivenoms. International Immunopharmacology. 2014; 21(1): 148–155. doi: 10.1016/j.intimp.2014.04.012 24815989

[9] Charlton T. King cobra: Natural history and captive management. Borneo: Natural history publication; 2018.

[10] Veto T, Price R, Silsby JF, & Carter JA. Treatment of the first known case of King Cobra envenomation in the United Kingdom, complicated by severe anaphylaxis. Anaesthesia. 2007;62(1): 75–78. doi: 10.1111/j.1365-2044.2006.04866.x 17156231

[11] Marshall BM, Strine CT, Jones MD, Theodorou A, Amber E, Waengsothorn S et al. Hits Close to Home: Repeated Persecution of King Cobras (Ophiophagus hannah) in Northeastern Thailand. Tropical Conservation Science. 2018;11. doi: 10.1177/1940082918818401

[12] Tan KY, Ng TS, Bourges A, Ismail AK, Maharani T, Khomvilai S et al. Geographical variations in King Cobra (Ophiophagus hannah) venom from Thailand, Malaysia, Indonesia and China: On venom lethality, antivenom immunoreactivity and in vivo neutralization. Acta Tropica. 2020;203: 105311. doi: 10.1016/j.actatropica.2019.105311 31862461

[13] Gowri Shankar P, Swamy P, Williams RC, Ganesh SR, Moss M, Höglund J et al. King or royal family? Testing for species boundaries in the King Cobra, Ophiophagus hannah (Cantor, 1836), using morphology and multilocus DNA analyses. Molecular Phylogenetics and Evolution. 2021; 165: 107300. doi: 10.1016/j.ympev.2021.107300 34474153

[14] Tan CH, Tan NH. Toxinology of Snake Venoms: The Malaysian Context. In Snake Venoms. 2015.

[15] Tan CH, Bourges A, Tan KY. King Cobra and snakebite envenomation: On the natural history, human-snake relationship and medical importance of Ophiophagus hannah. Journal of Venomous Animals and Toxins Including Tropical Diseases. 2021; 27: 1–19. doi: 10.1590/1678-9199-JVATITD-2021-0051 35069710 PMC8733962

[16] Ministry of Health M. Guideline: Management of Snakebite. Ministry of Health Malaysia. 2017.

[17] Jamaiah Ibrahim, Rohela M, Ng TK, Ch’ng KBH, Teh YS, Nurulhuda AL et al. Retrospective prevalence of snakebites from Hospital Kuala Lumpur (HKL) (1999–2003). Southeast Asian J Trop Med Public Health. 2006;37(1):200–5. 16771235

[18] Sharma N, Chauhan S, Faruqi S, Bhat P, Varma S. Snake envenomation in a north Indian hospital. Emerg Med J. 2005;22(2):118–20. doi: 10.1136/emj.2003.008458 15662063 PMC1726667

[19] Curry SC, Horning D, Brady P, Requa R, Kunkel DB, Vance MV. The legitimacy of rattlesnake bites in central Arizona. Ann Emerg Med. 1989;18(6):658–63. doi: 10.1016/s0196-0644(89)80523-2 2729691

[20] Reid HA, Thean PC, Artin WJ. Epidemiology of snake bite in north Malaya. Br Med J. 1963;1(5336):992–7. doi: 10.1136/bmj.1.5336.992 13973752 PMC2122840

[21] Le HQ, Nguyen NTT, Vo TNA, Van Nguyen T, Do KTN, Ho TTC, et al. Envenoming by king cobras (Ophiophagus hannah) in Vietnam with cardiac complications and necrotizing fasciitis. Toxicon. 2021;200:127–33. doi: 10.1016/j.toxicon.2021.07.007 34302855

[22] Gold BS, Pyle P. Successful treatment of neurotoxic king cobra envenomation in Myrtle Beach, South Carolina. Ann Emerg Med. 1998;32(6):736–8. doi: 10.1016/s0196-0644(98)70075-7 9832672

[23] Ganthavorn S. A case of king cobra bite. Toxicon. 1971;9(3):293–4. doi: 10.1016/0041-0101(71)90084-5 5092397

[24] Tin M, Rai M, Maung C, Tun P, Warrell DA. Bites by the king cobra (Ophiophagus hannah) in Myanmar: successful treatment of severe neurotoxic envenoming. Q J Med. 1991;80(293):751–62. 1754675

[25] Ministry of Health Malaysia. Pain as the 5th Vital Sign Guideline: 3rd Edition. Malaysia: Ministry of Health; 2018.

[26] Ismail AK, Cham G. Snake-related Injuries. In Ooi S, Low M, & Manning P (Eds.), Guide to the essentials in Emergency Medicine. 3rd ed. Singapore: Mcgraw Hill; 2022. pp. 622–631.

[27] Imran R, Vanat Q, Hausien O, Jose R. King cobra bite—Can early decompression prevent digital amputation? JPRAS Open. 2021;27:12–6. doi: 10.1016/j.jpra.2020.11.004 33299920 PMC7704406

[28] Wetzel WW, Christy NP. A king cobra bite in New York City. Toxicon. 1989;27(3):393–5. doi: 10.1016/0041-0101(89)90186-4 2728028

[29] Subedi N, Paudel IS, Khadka A, Shrestha U, Mallik VB, Ankur KC. Knowledge of first aid methods and attitude about snake bite among medical students: a cross sectional observational study. J Occup Med Toxicol. 2018;13:26. doi: 10.1186/s12995-018-0210-0 30147746 PMC6094924

[30] Bhaumik S, Beri D, Lassi Z, Jagnoor J. Interventions for the management of snakebite envenoming: An overview of systematic reviews. PLOS Neglected Tropical Diseases. 2020;14:e0008727. doi: 10.1371/journal.pntd.0008727 33048936 PMC7584233

[31] Patikorn C, Ismail AK, Abidin SAZ, Blanco FB, Blessmann J, Choumlivong K et al. Situation of snakebite, antivenom market and access to antivenoms in ASEAN countries. BMJ Global Health. 2022; 7(3), e007639. doi: 10.1136/bmjgh-2021-007639 35296460 PMC8928241

[32] Patikorn C, Blessmann J, Nwe MT, Tiglao PJG, Vasaruchapong T, Maharani T, et al. Estimating economic and disease burden of snakebite in ASEAN countries using a decision analytic model. PLoS Negl Trop Dis. 2022;16(9):e0010775. doi: 10.1371/journal.pntd.0010775 36170270 PMC9518918

[33] Patikorn C, Ismail AK, Zainal Abidin SA, Othman I, Chaiyakunapruk N, Taychakhoonavudh S. Potential economic and clinical implications of improving access to snake antivenom in five ASEAN countries: A cost-effectiveness analysis. PLoS Negl Trop Dis. 2022;16(11):e0010915. doi: 10.1371/journal.pntd.0010915 36383562 PMC9668136

[34] Muthusamy E. Snake bite: experience in Bukit Mertajam District Hospital, Pulau Pinang. Singapore Med J. 1988;29(4):383–6. 3249968

[35] Ranawaka UK, Lalloo DG, de Silva HJ. Neurotoxicity in snakebite—the limits of our knowledge. PLoS Negl Trop Dis. 2013;7(10):e2302. doi: 10.1371/journal.pntd.0002302 24130909 PMC3794919

[36] Smith TA 2nd, Figge HL. Treatment of snakebite poisoning. Am J Hosp Pharm. 1991;48(10):2190–6. 1781479

[37] Chen FC, Ismail AK, Mao YC, Hsu CH, Chiang LC, Shih CC, et al. Application of Sonographic Assessments of the Rate of Proximal Progression to Monitor Protobothrops mucrosquamatus Bite-Related Local Envenomation: A Prospective Observational Study. Trop Med Infect Dis. 2023;8(5). doi: 10.3390/tropicalmed8050246 37235294 PMC10223384

[38] Chen HL, Chuah YQ, Eng KL, Michelle YYL, Ahmad R. Use of Snake Antivenom and Clinical Outcomes in Snake Envenomation: A Retrospective Study in a Tertiary Hospital in Penang, Malaysia. Asia Pacific Journal of Medical Toxicology. 2019;8:78–82. doi: 10.22038/APJMT.2019.13797

[39] Pichler WJ, Adkinson NF, Feldweg AM. An approach to the patient with drug allergy. UpToDate. 2016;24(3):151–72. Available from: https://www.uptodate.com/contents/an-approach-to-the-patient-with-drug-allergy#

[40] Campbell RL, Kelso JM. Anaphylaxis: acute diagnosis. UpToDate. 2016. https://www.uptodate.com/contents/anaphylaxis-acute-diagnosis?topicRef=392&source=see_link#topicContent

[41] Rowe BH, Grunau B. Allergy and Anaphylaxis. In Tintinalli J. E. (Ed.), Emergency medicine: A comprehensive study guide. USA: Mc Graw Hill. 2020. pp. 68–71.

[42] Singer E, Zodda D. Allergy And Anaphylaxis: Principles Of Acute Emergency Management. Emerg Med Pract. 2015;17(8):1–19; quiz 20. 26237051

[43] Amin M, Mamun SMH, Rashid R, Rahman M, Ghose A, Sharmin S, et al. Anti-snake venom: Use and adverse reaction in a snake bite study clinic in Bangladesh. Journal of Venomous Animals and Toxins Including Tropical Diseases—J Venom Anim Toxins Trop Dis. 2008;14. doi: 10.1590/S1678-91992008000400009

[44] Seneviratne SL, Opanayaka CJ, Ratnayake NS, Kumara KE, Sugathadasa AM, Weerasuriya N, et al. Use of antivenom serum in snake bite: a prospective study of hospital practice in the Gampaha district. Ceylon Med J. 2000;45(2):65–8. doi: 10.4038/cmj.v45i2.8003 11051703

[45] Hammerberg EM, Bachur RG. Acute compartment syndrome of the extremities. UpToDate. 2022. https://www.uptodate.com/contents/acute-compartment-syndrome-of-the-extremities#topicContent

[46] Ho CH, Ismail AK, Liu SH, Tzeng YS, Li LY, Pai FC, et al. The role of a point-of-care ultrasound protocol in facilitating clinical decisions for snakebite envenomation in Taiwan: a pilot study. Clin Toxicol (Phila). 2021;59(9):794–800. doi: 10.1080/15563650.2021.1881535 33605805

[47] Wood D, Sartorius B, Hift R. Ultrasound findings in 42 patients with cytotoxic tissue damage following bites by South African snakes. Emerg Med J. 2016;33(7):477–81. doi: 10.1136/emermed-2015-205279 27068867

[48] Bhatia N, Castro-Borobio M, Greene JN, Nanjappa S. Necrotizing Fasciitis Secondary to Aeromonas Infection Presenting with Septic Shock. Case Rep Med. 2017;2017:4607582. doi: 10.1155/2017/4607582 29081807 PMC5610789

[49] Lota AS, Altaf F, Shetty R, Courtney S, McKenna P, Iyer S. A case of necrotising fasciitis caused by Pseudomonas aeruginosa. J Bone Joint Surg Br. 2010;92(2):284–5. doi: 10.1302/0301-620X.92B2.22688 20130324

[50] Dehghani R, Sharif MR, Moniri R, Sharif A, Haddad Kashani H. The identification of bacterial flora in oral cavity of snakes. Comparative Clinical Pathology. 2016;25. doi: 10.1007/s00580-015-2178-9

[51] Panda S, Padhi L, Sahoo G. Oral bacterial flora of Indian cobra (Naja naja) and their antibiotic susceptibilities. Heliyon. 2018;4:1008. doi: 10.1016/j.heliyon.2018.e01008 30582036 PMC6298943

[52] Knudsen C, Jürgensen JA, Føns S, Haack AM, Friis RUW, Dam SH, et al. Snakebite Envenoming Diagnosis and Diagnostics. Front Immunol. 2021;12:661457. doi: 10.3389/fimmu.2021.661457 33995385 PMC8113877

