# Supplementary material for: Frequency, geographical distribution, clinical characteristics, antivenom utilisation and outcomes of King Cobra (Ophiophagus hannah) bites in Malaysia
Source: PLoS Negl Trop Dis. 2024 Jul 25;18(7):e0012359. doi: 10.1371/journal.pntd.0012359 (PMC11302923; doi:10.1371/journal.pntd.0012359)
Supplement: S1 Data — (DOCX) [file pntd.0012359.s001.docx]

**Table S1. Supplementary minimal dataset** **of each confirmed King Cobra bite incident location, demographics, amount of antivenom usage and days of hospital stay.**

| **Case ID *n*=32** | **RECS Consultation Groups** | **Age (yr)** | **Gender (M/F)** | **Nationality (M/NM)** | **Time of Incident** | **Total AV (Vials)** | **Hosp (Days)** |
| --- | --- | --- | --- | --- | --- | --- | --- |
| 2015.1 | Borneo | 28 | M | NM | 1730 | 30 | 14 |
| 2015.2 | Kemaman | 21 | M | M | 1930 | 0 | 0 |
| 2015.3 | Kuala Lipis | 31 | M | M | 1230 | 0 | 2 |
| 2015.4 | Ipoh | 55 | M | M | 1700 | 25 | 0 |
| 2015.5 | Kemaman | 26 | M | M | 1930 | 20 | 8 |
| 2015.6 | Johor | 30 | M | M | 1545 | 20 | 9 |
| 2015.7 | Serdang | 37 | F | M | 1445 | 0 | 1 |
| 2016.1 | Sg Buloh | 32 | M | M | 1840 | 0 | 1 |
| 2016.2 | Kuantan | 20 | M | M | 1900 | 0 | NA |
| 2016.3 | Muar | 25 | M | M | 1340 | 10 | 2 |
| 2017.1 | Gerik&Ipoh | 45 | M | M | 1800 | 10 | 5 |
| 2017.2 | Taiping, Parit Buntar | 69 | M | M | 1700 | 10 | 7 |
| 2017.3 | Melaka | 77 | M | M | 1600 | 25 | 4 |
| 2017.4 | Kuantan | 20 | M | M | 1200 | 5 | 2 |
| 2017.5 | Johor | 1 | F | M | 1800 | 0 | 1 |
| 2017.6 | Temerloh Jerantut | 32 | M | M | 1000 | 0 | 1 |
| 2018.1 | Sarawak Central | 13 | M | M | 1430 | 0 | 2 |
| 2018.2 | Kuantan | 30 | M | M | 0130 | 10 | 2 |
| 2018.3 | Bentong&Temerloh | 32 | M | M | 2030 | 25 | 4 |
| 2018.4 | HKL | NA | M | M | 2350 | 10 | 3 |
| 2018.5 | Sarawak Central | 74 | M | M | 1200 | 0 | 1 |
| 2019.1 | Taiping, Parit Buntar | 24 | M | M | 0950 | 8 | NA |
| 2019.2 | Johor | 22 | M | NM | 1200 | 8 | NA |
| 2019.3 | Perlis | 50 | M | M | 1300 | 10 | 5 |
| 2019.4 | Melaka | 23 | M | M | 1130 | 10 | 3 |
| 2019.5 | Perlis | 26 | M | M | 1600 | 8 | NA |
| 2019.6 | Taiping, Parit Buntar | 71 | M | M | 1800 | 20 | 5 |
| 2020.1 | Kuantan | 34 | M | M | 1840 | 0 | NA |
| 2020.2 | Ampang | 31 | M | M | 2200 | 20 | 5 |
| 2020.3 | Penang | 19 | M | M | 1945 | 15 | 6 |
| 2020.4 | Dungun&Terengganu | 23 | M | M | 2300 | 8 | 5 |
| 2020.5 | HSAH, Yan Baling Sik | 51 | M | M | 1100 | 10 | 3 |


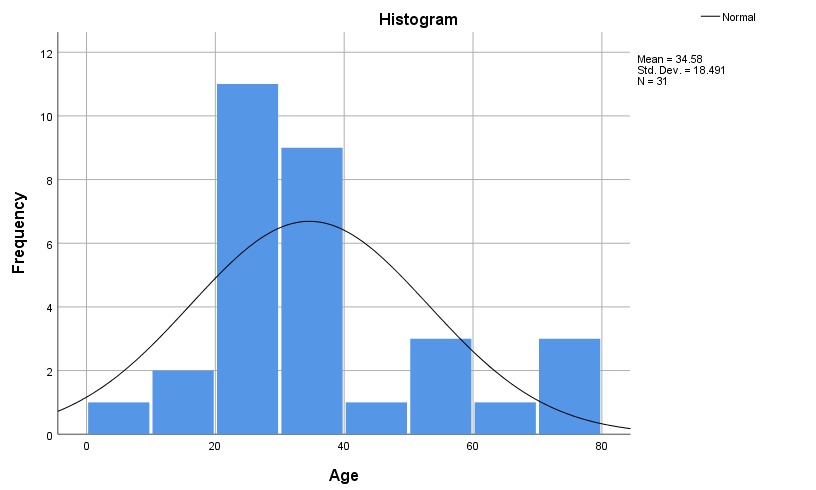


The mean age was 34.58 years old (SD ±18.5)


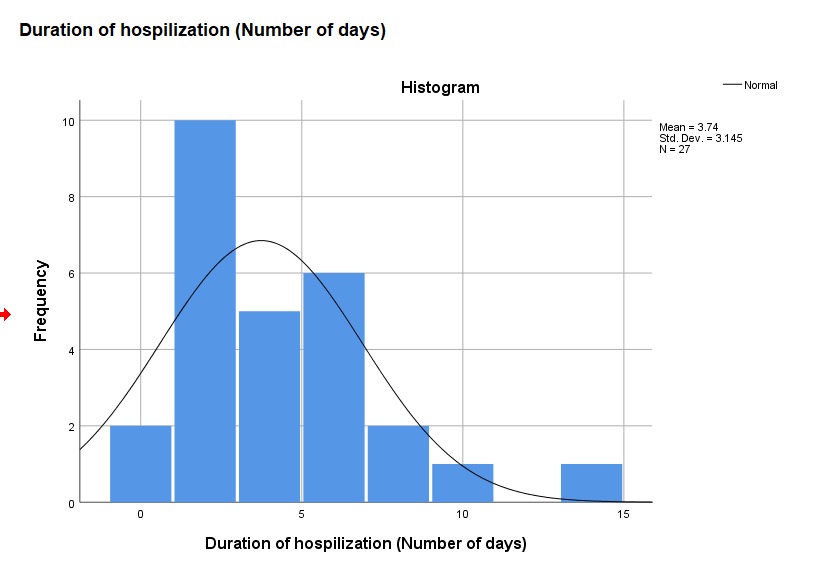


The median length of stay was 3 (IQR 1-5)
